# Role of S100A8/A9 in Platelet–Neutrophil Complex Formation during Acute Inflammation

**DOI:** 10.3390/cells11233944

**Published:** 2022-12-06

**Authors:** Julian Revenstorff, Nadine Ludwig, Annika Hilger, Sina Mersmann, Martin Lehmann, Julia Chiara Grenzheuser, Marina Kardell, Julia Bone, Niklas Martin Kötting, Nina Christine Marx, Johannes Roth, Thomas Vogl, Jan Rossaint

**Affiliations:** 1 Institute of Immunology, University Hospital Münster, 48149 Münster, Germany; 2 Department of Anesthesiology, Intensive Care and Pain Medicine, University Hospital Münster, 48149 Münster, Germany

**Keywords:** MRP8/14, calprotectin, platelets, neutrophils, platelet–neutrophil complexes, acute inflammation, ARDS

## Abstract

Acute respiratory distress syndrome (ARDS) due to pulmonary infections is associated with high morbidity and mortality. Upon inflammation, the alarmin S100A8/A9 is released and stimulates neutrophil recruitment mainly via binding to Toll-like receptor 4 (TLR4). TLR4 is also expressed on platelets, which modulate the immune response through direct interaction with leukocytes. In a murine model of *Klebsiella pneumoniae*-induced pulmonary inflammation, global S100A9 deficiency resulted in diminished neutrophil recruitment into the lung alveoli and neutrophil accumulation in the intravascular space, indicating an impaired neutrophil migration. A lack of TLR4 on platelets resulted in reduced neutrophil counts in the whole lung, emphasising the impact of TLR4-mediated platelet activity on neutrophil behaviour. Flow cytometry-based analysis indicated elevated numbers of platelet–neutrophil complexes in the blood of S100A9^−/−^ mice. Intravital microscopy of the murine cremaster muscle confirmed these findings and further indicated a significant increase in neutrophil–platelet complex formation in S100A9^−/−^ mice, which was reversed by administration of the S100A8/A9 tetramer. An in vitro bilayer assay simulated the murine alveolar capillary barrier during inflammation and validated significant differences in transmigration behaviour between wild-type and S100A9^−/−^ neutrophils. This study demonstrates the role of S100A8/A9 during platelet–neutrophil interactions and neutrophil recruitment during pulmonary inflammation.

## 1. Introduction

Acute pulmonary inflammation is characterised by increased permeability of pulmonary capillary endothelial cells and alveolar epithelial cells [1,2]. It is precipitated by direct pulmonary insults such as bacterial pneumonia and aspiration of gastric content or indirect systemic inflammatory reactions in response to sepsis, transfusion, or major trauma [1,3,4]. Acute respiratory distress syndrome (ARDS) is characterised by severe lung inflammation and polymorphonuclear leukocyte (PMN, neutrophil) recruitment into the inflamed tissue, deposition of hyaline membranes and alveolar-capillary barrier damage. Increased permeability due to disruption of the endothelial and epithelial layer results in pulmonary oedema formation and compromised gas exchange, resulting in severe arterial hypoxaemia and impaired carbon dioxide excretion [1,2,5]. Preclinical models have suggested that direct lung injury starts with an insult to the lung epithelium, but indirect lung injury originates with systemic endothelial damage due to inflammatory mediators [6]. Even though therapeutic approaches, including lung-protective ventilation and fluid-conservative management, have reduced mortality and morbidity, the development of pharmacological therapies remains challenging due to the complexity of the syndrome and the broad clinical phenotype [1].

Platelets are an integral part of primary haemostasis, but are also actively involved in immune processes [7]. Platelets and PMNs interact indirectly via the release of proinflammatory mediators and directly via physical interaction to form platelet–neutrophil complexes (PNC) [8]. Platelets are a source of chemokines such as PF4, RANTES and CXCL7, which are involved in PMN recruitment and activation [9,10,11]. Platelets further release thromboxane A_2_, which induces upregulation of ICAM-1 and thereby indirectly promotes PMN recruitment to the lung [3]. Direct physical interaction is dependent on different adhesion molecules. PNC formation is initiated by the binding of P-selectin (CD62P) on the platelet surface to PSGL-1 on PMNs. Firmer adhesion is enabled by binding of Mac-1 to platelet GPIb or GPIIbIIIa via a bridge of fibrinogen [9,12]. Subsequent outside-in signaling events facilitate PMN activation, migration and effector functions such as NET formation [8,11,13]. Platelet–neutrophil aggregates have been found in the lung microvasculature during inflammation and acute lung injury [10,14,15]. Blocking P-selectin, and thereby PNC formation, has been shown to reduce PMN recruitment into the lung and vascular permeability, as well as improve gas exchange during acid-induced lung injury [16]. However, an important role for platelet–neutrophil aggregates has not only been suggested for sterile inflammation but also for systemic inflammation caused by sepsis-relevant pathogens, as *Klebsiella pneumoniae* and *Staphylococcus aureus* enhance the formation of PNCs [17]. Furthermore, an infection with SARS-CoV-2 {XE “SARS-CoV-2” \t “*Severe acute respiratory syndrome related coronavirus 2*”} is associated with increased platelet–leukocyte aggregates and proinflammatory priming of platelets, which is associated with the release of increased amounts of cytokines, chemokines and growth factors upon stimulation [18].

S100A8 and S100A9 (also known as MRP8 and MRP14, respectively) are Ca^2+^-binding proteins belonging to the S100 family. Human S100A8 and S100A9 consist of 93 and 113 amino acid residues, respectively, and S100A9 has a truncated isoform with 110 amino acids, and both isoforms can be phosphorylated by p38 MAP kinase [19]. S100A8 and S100A9 form homo- and heterodimer complexes upon release from activated phagocytes and may induce a broad spectrum of proinflammatory effects [20]. We could previously show that the physiologically relevant forms of S100A8 and S100A9 are heterodimers [21]. The activity of a heterodimer released by phagocytes must be limited in time by calcium-induced tetramerisation to avoid systemic overreactions of the immune system [21]. Therefore, the activity of heterodimers is short-lived, locally restricted and abrogated if two heterodimers form a stable tetramer driven by extracellular calcium concentrations. Tetramerisation hides the TLR4-binding site in the tetramer interphase, and mutations in the heterodimer that inhibit tetramerisation lead to an uncontrolled activity. Current work demonstrates the inhibitory, calming effect of S100A8/A9 tetramer on monocytes mediated by the receptor CD69 [22]. Platelets also express CD69, yet the effect of the S100A8/A9 tetramer on platelets and PNC formation is still unknown. The aim of this study was the investigation of the role of S100A8/A9 proteins in PNC formation, neutrophil recruitment and the fate of pulmonary inflammation in murine models of inflammation in vivo and in vitro.

## 2. Materials and Methods

### 2.1. Animals and Reagents

Nine- to twenty-week-old male and female wild-type (WT), S100A9^−/−^, TLR4^Pf4Cre-^ and TLR4^Pf4Cre+^ mice of a C57BL/6 background were used throughout this study. Mice were maintained in a barrier facility under specific pathogen-free (SPF) conditions with food and water ad libitum. All mouse experiments were approved by the North Rhine-Westphalia Office for Nature, Environment and Consumer Protection (LANUV NRW, reference number 81-02.04.2019.A445). Recombinant S100A8/A9 protein was produced in-house at the Institute of Immunology, University of Münster, as described previously (Vogl, JCI, 2018).

### 2.2. Klebsiella pneumoniae-Induced Lung Infection

Age- and sex-matched mice were infected with viable *Klebsiella pneumoniae* and neutrophil recruitment to the lung was analysed after 22 h, as described in Rossaint et al. [23]. Briefly, *K. pneumoniae* (ATCC, strain 13883) were grown overnight in tryptic soy broth, and washed and resuspended in sterile PBS at a final concentration of 8 × 10^8^ cells/mL. Mice were anaesthetised by intraperitoneal injection of ketamine (125 µg/g body weight; WDT) and xylazine (12.5 µg/g body weight; Bayer, Leverkusen, Germany) and challenged with 4 × 10^7^ *K. pneumoniae* per mouse via intratracheal injection. For S100A8/A9 tetramer supplementation experiments, 150 µg of S100A8/A9 was injected into the peritoneum immediately after infection, and again after 11 h. All mice survived the observation period and were sacrificed after 22 h. Blood was drawn via cardiac puncture and the lungs were lavaged four times with 0.7 mL each of a physiologic saline solution (NaCl 0.9%, B. Braun, Melsungen, Germany). The lung circulation was perfused with 3 mL PBS and lungs were enzymatically digested with DNase I, hyaluronidase type I-s and collagenase type XI (all Sigma-Aldrich). The number of transmigrated neutrophils in the bronchoalveolar lavage fluid (BALF) and the lung tissue was determined via Kimura staining and subsequent CD45/Ly-6B.2/Gr-1 (CD45-PerCP/Cy5.5 (clone 30/F11, BioLegend, San Diego, CA, USA), Ly-6B.2-FITC (clone 7/4, Bio-Rad, Hercules, CA, USA), Gr-1-AF633 (clone RB6-8C5, purified from hybridoma supernatant)) antibody staining for flow cytometry-based analysis (FACSCantoII, BD Bioscience, Heidelberg, Germany). CFUs in the BALF were determined by serial plating on tryptic soy agar plates. 

### 2.3. Luminex Analysis of Inflammatory Mediators

Blood samples from healthy and *K. pneumoniae* infected mice were centrifuged for 10 min, at 2000× *g* and 4 °C. The plasma phase was collected and analysed using the Cytokine & Chemokine 36-Plex Mouse ProcartaPlex Panel Kit 1A (Thermo Fisher Scientific, Waltham, MA, USA) run on a Luminex 200 analyser (Luminex Corp., Austin, TX, USA) according to the manufacturer’s instructions. 

### 2.4. Isolation of Murine Lung Microvascular Endothelial Cells

Murine lung microvascular endothelial cells (MLMVECs) were isolated from 15–20-week-old WT mice, as described previously [24]. Briefly, mice were sacrificed via cervical dislocation and lungs were removed, minced and digested with 20 mg/mL collagenase A (Roche) at 37 °C for 90 min. Lung homogenates were incubated with Dynabeads (Life Technologies) coupled with an anti-CD31 antibody (clone Mec13.3, BD) for 45 min at 4 °C. Dynabead-bound MLMVECs were isolated from CD31-negative cells via magnetic separation. MLMVECs were cultured on gelatine-coated dishes at 37 °C and 10% CO_2_. 

### 2.5. Isolation of Murine Alveolar Epithelial Cells Type II

Alveolar epithelial cells (AECs) type II were isolated according to a previous publication [5]. Fifteen-to-twenty-week-old WT mice were anaesthetised via intraperitoneal injection of ketamine (125 µg/g body weight; WDT pharmaceuticals) and xylazine (12.5 µg/g body weight; Bayer). The lung circulation was perfused with 10 mL PBS (Roth) and the trachea was exposed. A total of 1 mL dispase (5000 U, Corning) was intratracheally injected into the lung with the help of a 22G angiocatheter followed by 0.5 mL of a 1% low-melt agarose. Whole lung lobes were removed and incubated in 0.5 mL dispase for 45 min at 25 °C with mild agitation. Subsequently, lung lobes were dissected and the cell suspension was serially filtered through 70 µm, 40 µm and 20 µm cell strainers. After erythrocyte lysis, CD45-positive (clone 30-F11, BD) and CD16/32-positive (clone 2.4G2) cells were magnetically separated from the cell suspension by using Streptavidin MagneSphere paramagnetic particles (Promega). AECs were cultured on fibronectin-coated dishes at 37 °C and 5% CO_2_. 

### 2.6. Bilayer Transmigration Assay

To prepare an alveolus-like in vitro bilayer construct, tTranswell^TM^ inserts (5 µm pores, Corning) were coated with 100 µg/mL fibronectin (Sigma-Aldrich). Subsequently, 4.5 × 10^4^ isolated type II AECs were seeded onto the underside of the Transwell^TM^, while 6 × 10^4^ MLMVECs were seeded on the Transwell^TM^ filter’s top side the next day. Confluent layers were obtained after four days of incubation at 37 °C and 5% CO_2_. On the day of transmigration, 4.5 × 10^5^ bone marrow-derived neutrophils, isolated via Pancoll (PAN Biotech) density gradient centrifugation, and 4.5 × 10^6^ washed platelets, isolated via repetitive slow-speed centrifugation [23], were gently mixed and added per Transwell^TM^ in a plasma-supplemented transmigration medium. Where indicated, 0.1 U/mL thrombin was added to the upper chamber. Cells were allowed to transmigrate through the bilayer for 3 h at 37 °C, 5% CO_2_ with mild agitation (80 rpm) following a CXCL1 gradient (100 ng/mL, Peprotech) applied to the lower Transwell^TM^ chamber. Afterwards, supernatants were collected from the upper and lower Transwell^TM^ chambers and cell counts were determined using a Sysmex haemocytometer. For visualisation and microscopical analyses of the Transwell^TM^, membranes were fixated, blocked and separated from the inlays with a scalpel. The bilayer was stained with CD41-BV421 (clone MWReg30, BioLegend), Gr-1-AF488 (clone RB6-8C5, purified from hybridoma supernatant), CD31-AF568 (clone Mec13.3, BioLegend), Podoplanin-AF647 (clone Pmab-1, BioLegend) in 1% BSA/PBS for 30 min at RT. Z-stacks were recorded using a confocal microscope (Carl Zeiss, CellObserver SD, Göttingen, Germany) equipped with a 20× objective (laser power and exposure time: BV421: 50%, 150 ms; AF488: 50%, 150 ms; AF568: 85%, 200 ms; AF647: 85%, 200 ms) and analysed with ImageJ and Imaris software. A total of 3 z-stacks per condition were analysed and the number of counted cells was extrapolated to the total area of the bilayer, which equals an area of 0.33 cm^2^. 

### 2.7. ImageStream Analysis

Blood from healthy mice was drawn via cardiac puncture with a heparinised syringe. The blood was diluted 1:2 with PBS and immediately stained with fluorescent antibodies (CD41-BV421, clone MWReg30, BioLegend; CD45-PE, clone 30-F11, BioLegend; Ly-6B.2-FITC, clone 7/4, Bio-Rad) in 1:200 dilution. Samples were stimulated simultaneously with 1 µM ADP (Sigma-Aldrich) where indicated. After 20 min, the reaction was stopped by fixing the samples in 2% PFA (Roth) and 15 min later diluted again 1:10 in PBS and subsequently analysed via imaging flow cytometry using the AMNIS^®^ ImageStream^®^ II (Luminex Corporation, Austin, TX, USA).

### 2.8. In Vitro Platelet–Neutrophil Complex Formation

Platelets and bone marrow neutrophils were isolated as described above. Platelets and neutrophils were combined at a 10:1 ratio in a final volume of 100 µL RPMI (PAN) containing 0.5% FCS (PAN) and 25 mM HEPES (Sigma). The cells were stimulated with 10 µM fMLP (Sigma) and 0.1 U/mL thrombin (Sigma) for 10 min at RT with subsequent antibody staining (CD41-BV421 (clone MWReg30, BioLegend), Gr-1-AF633 (clone RB6-8C5, purified from hybridoma supernatant), CD45-PE (clone 30-F11, BioLegend), Ly6B.2-FITC (clone 7/4, Bio-Rad)). CD41^+^ signals were compared to isotype controls (BV421 Rat IgG1, κ isotype ctrl antibody, BioLegend) and measurements were performed with a FACSCantoII flow cytometer. 

### 2.9. Intravital Microscopy of the Murine Cremaster Muscle

Intravital microscopy of the murine cremaster muscle was performed as previously described [25,26]. Briefly, mice received an intrascrotal injection of 500 ng TNFα (BioLegend) in 0.3 mL saline two hours before intravital microscopy. For S100A8/A9 tetramer supplementation experiments, 100 µg of S100A8/A9 was added to the TNFα injection. For fluorescent labelling of platelets and neutrophils, mice were treated with CD41-BV421 (clone MWReg30, BioLegend) and Gr-1-AF488 (clone RB6-8C5, purified from hybridoma supernatant) antibody suspension via intravenous injection right before anaesthesia (ketamine/xylazine) and cremaster muscle exteriorisation. High-speed multichannel fluorescence microscopy was performed on an upright microscope (Axioskop; Carl Zeiss, Göttingen, Germany) equipped with a Lambda DG-4 ultra-high-speed wavelength switcher (Sutter Instruments, Novato, CA, USA) and a 40 × 0.75 NA saline immersion objective. Videos were recorded with a digital camera (Sensicam QE) and analysed with Slidebook Software (Version 5; Intelligent Imaging Innovations, Göttingen, Germany). All measurements were performed in postcapillary venules with a diameter between 20 and 40 µm. Transmigration was analysed using reflected light oblique transillumination microscopy (RLOT) and the number of extravasated leukocytes was determined in an area of 75 × 100 µm to each side of a vessel [27]. 

### 2.10. Western Blot Analysis

Platelets and bone marrow-derived neutrophils were isolated and lysed with RIPA buffer. Lysates were heated at 95 °C for 10 min and subjected to Western blotting according to standard procedures (Bio-Rad, mini-PROTEAN Tetra Cell System). Membranes were blocked in TBST + 5% milk powder (Roth) for 1 h at RT. Subsequently, membranes were incubated overnight with antimurine S100A8 and S100A9 antibodies (Abbiotec). Anti-GAPDH immunobands (Cell Signalling) were used as loading control. The ECL system (GE Healthcare) was used for development of the immunoblots. Analysis of Western blots was performed using the ChemiDoc device (Bio-Rad).

### 2.11. Flow Cytometry

For the analysis of TLR4/MD2 expression on murine platelets, platelets were isolated from TLR4^PF4Cre-^ and TLR4^PF4Cre+^ mice via repetitive slow-speed centrifugation and stained with CD41-BV421 (clone MWReg30, BioLegend) and TLR4/MD2-APC (clone MTS510, Thermo Fisher Scientific) antibodies for 20 min at RT. Expression levels were investigated in a FACSCantoII flow cytometer (BD Bioscience). Stimulation of isolated WT platelets with S100A8/A9 tetramers was performed according to Colicchia et al. with minor modifications [28]. Murine platelets were isolated via repetitive slow-speed centrifugation and resuspended in Tyrode’s buffer (20 mM HEPES, 136 mM NaCl, 2.6 mM KCl, 12 mM NaHCO_3_, 5.5 mM D-glucose, 2 mM CaCl_2_, 1 mM MgCl_2_). Platelets (2 × 10^6^) were stimulated with 40 µg/mL S100A8/A9 (produced and purified by T. Vogl). CD62P mobilisation and GPIIbIIIa activation were assessed by antibody staining (CD62P-PE, clone RMP-1, BioLegend; act. GPIIbIIIa-PE, clone JON/A, Emfret Analytics) and analysed with FlowJo software. 

### 2.12. Surface Marker Expression

Surface expression of proteins on neutrophils and platelets was assessed in murine whole blood. Mice were anaesthetised via intraperitoneal injection of ketamine (125 µg/g body weight; WDT) and xylazine (12.5 µg/g body weight; Bayer, Leverkusen, Germany) and blood was drawn via cardiac puncture into an ACD (Sigma-Aldrich)-coated syringe with a 27G cannula. Blood was diluted 1:5 with PBS. As a low stimulus, 1 µM ADP (Sigma-Aldrich) was added, while controls were left untreated. Diluted full blood was stained with CD45-APC/Cy7 (clone 30-F11, BioLegend), Ly-6G-AF488 (clone 1A8, BioLegend), CD41-BV421 (clone MWReg30, BioLegend), CD11a-PE (clone M7/14, BioLegend), CD11b-PE (clone M1/70, BD Bioscience), CD162-PE (clone 2PH1, BD Bioscience), CD40-PE (clone FGK45, BioLegend), GP1bα-AF488 (clone Xia.G5, Emfret Analytics, Würzburg, Germany), Ly49Q mAb-PE (clone JON/A, emfret), CD154-PE (clone MR1, BioLegend), CD62P-PE (clone RMP-1, BioLegend) and CD102-AF488 (clone 3C4, BioLegend). Neutrophils were considered CD45 + Ly-6G+ populations, while platelets were identified as CD45-CD41+.

### 2.13. Fibrinogen ELISA and Blood Cell Counts

The amount of fibrinogen in plasma samples was analysed with a murine fibrinogen ELISA (abcam) according to the manufacturer’s instructions. Blood was drawn via cardiac puncture and coagulation was counteracted with heparin (ratiopharm). Blood cell counts were analysed using the Sysmex haemocytometer and the remaining blood was centrifuged for 10 min, at 2000× *g* and 4 °C. The plasma phase was collected and stored at −20 °C until ELISA analysis. 

### 2.14. Statistics

Statistical analysis was performed with GraphPad PRISM (version 9). Data distribution was assessed using Kolmogorov–Smirnov-test or Shapiro–Wilk test. Two groups were compared using Student’s *t*-test or Mann–Whitney rank-sum test as appropriate. Multigroup analyses were performed using one-way ANOVA, and two-way ANOVA was applied for more than two groups with two different categorical independent variables, all followed by post hoc testing. All data are represented as mean ± SD. A *p*-value < 0.05 was considered statistically significant. For in vivo experiments, the provided *n* is the number of animals used per experiment.

## 3. Results

### 3.1. Neutrophil Response Is Hampered in S100A9^−/−^ and TLR4^PF4Cre+^ Mice during Pneumonia

**Figure 1:**
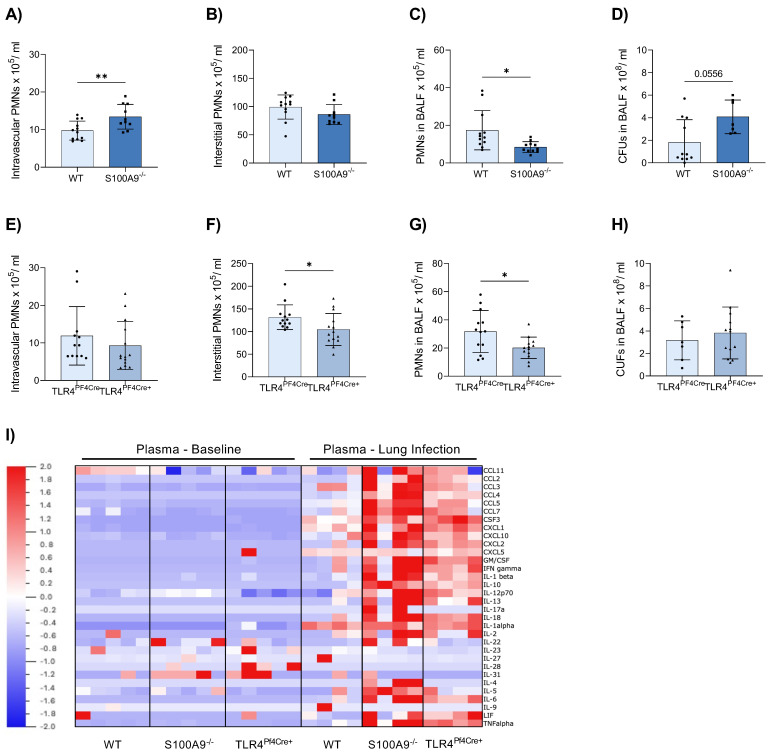
S100A9 deficiency and conditional knockout of TLR4 on platelets result in diminished neutrophil recruitment during acute inflammation. S100A9^−/−^ and TLR4^PF4Cre+^ mice were challenged with 40 × 10^6^ *K. pneumoniae* via intratracheal injection to induce acute pneumonia. Mice were sacrificed after 22 h, and neutrophil (PMN) recruitment as well as colony-forming units (CFUs) were assessed in comparison to appropriate WT controls. Neutrophil counts in the intravascular (**A**,**E**), interstitial (**B**,**F**) and alveolar space (BALF, bronchoalveolar lavage fluid) (**C**,**G**) were determined by flow cytometry. CFUs were analysed in the bronchoalveolar fluid lavage (**D**,**H**). Plasma samples from healthy and infected mice were analysed for 36 pro- and anti-inflammatory cytokines in a ProcartaPlex Panel Kit. Results for each individual mouse are presented as a heat map (**I**). Data are mean ± SD, n = 7–12, * *p* < 0.05, ** *p* < 0.005, student *t* test.

Several studies demonstrated that S100A8/A9 affects the outcome of bacterial infections. To analyse the effects of S100A8/A9 and its counterreceptor TLR4 on platelets during acute pneumonia, we examined neutrophil recruitment in global S100A9^−/−^ and conditional TLR4^PF4Cre+^ mice upon intratracheal infection with the clinically relevant pathogen *K. pneumoniae*. Of note, S100A9^−/−^ mice are almost completely double-deficient for S100A9 as well as S100A8 on the protein level, indicating a S100A8/S100A9 double-deficient mouse [29]. In comparison with appropriate WT controls, we observed a significant increase in neutrophil numbers in the intravascular compartment in S100A9^−/−^ (Figure 1A), which was not apparent in TLR4^PF4Cre+^ mice (Figure 1E). However, neutrophil recruitment to the interstitium was significantly reduced in TLR4^PF4Cre+^ mice (Figure 1F) but equal in S100A9^−/−^ mice compared to controls (Figure 1B). Both S100A9^−/−^ and TLR4^PF4Cre+^ mice demonstrated a significantly decreased neutrophil abundance in the bronchoalveolar lavage fluid (BALF, Figure 1C,G). Hampered neutrophil recruitment in S100A9^−/−^ mice was associated with a considerably elevated, albeit not statistically significant, bacterial burden (Figure 1D), which was less pronounced in TLR4^PF4Cre+^ mice compared to WT controls (Figure 1H). 

In addition to the analysis of neutrophil recruitment and bacterial spreading, we also examined plasma levels of inflammatory cytokines upon infection (Figure 1I, Supplementary Figure S1). Particularly in S100A9^−/−^, and to a lesser extent in TLR4^PF4Cre+^ mice, cytokine levels were strongly increased during pneumonia compared to WT controls. The increase in cytokine levels may be due to the increased number of neutrophils that were unable to leave the circulation. Since these cells fail to reach their target sites, there is also no negative feedback stopping the production of cytokines. These data are congruent with previously reported findings that elude to a faulty recruitment of neutrophils to the lung of S100A9^−/−^ mice after pneumonia challenges with other bacteria [30]. The lack of recruitment of neutrophils to the lung in both S100A9^−/−^ and TLR4^PF4Cre^ mice suggests an important role for platelets and platelet TLR4 in immune cell targeting. 

### 3.2. Platelets Do Not Express S100A8/A9

In the past, platelets have been described as sources of endogenous S100A8/A9 [31]. Since this would be a significant confounding factor for our analyses, we examined S100A8/A9 protein expression in platelets by Western blot analysis. Here, we demonstrate that platelets do not express S100A8/A9 on their own (Supplementary Figure S2A). Additionally, we confirm the expression of the TLR4/MD2 receptor on the surface of platelets (Supplementary Figure S2B), as has been described previously [32]. Recent studies have shown that high concentrations of S100A8/A9 are able to induce direct platelet activation via TLR4 and GPIbα [28,33]. Since TLR4 is known as the counterreceptor to S100A8/A9 heterodimers, we stimulated platelets with mutated S100A8/S100A9N70AE79A heterodimers unable to form tetramers, and could confirm GPIIbIIIa—but not CD62P—upregulation (Supplementary Figure S2C). 

### 3.3. Platelets Do Not Cross the Endothelial–Epithelial Bilayer during Transmigration in an In Vitro Bilayer Model

**Figure 2:**
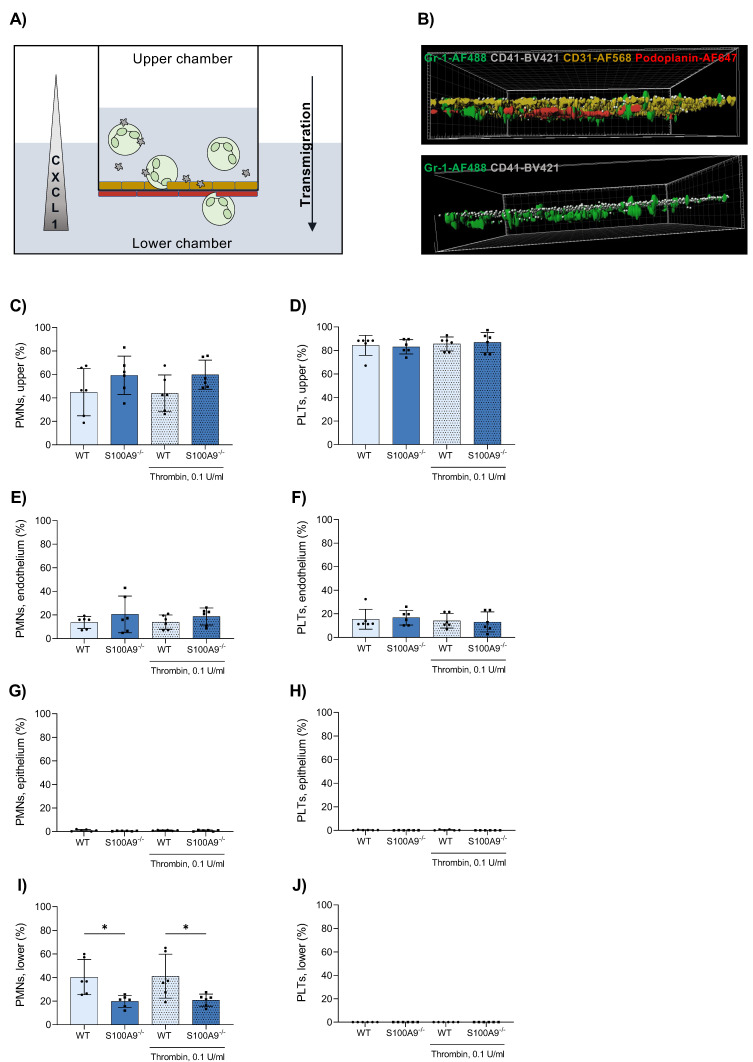
An in vitro murine bilayer model of primary alveolar endothelial and epithelial cells mimics the alveolar environment. Primary alveolar epithelial and endothelial cells were cultured on different sides of a Transwell^TM^ insert. (**A**) Platelets and bone marrow-derived neutrophils (PMNs) were posited in the upper chamber and allowed to transmigrate along a CXCL1 gradient for 3 h at 37 °C with mild agitation (neutrophils = green, platelets = grey, endothelial cells = yellow, epithelial cells = red). After transmigration, Transwell^TM^ membranes were cut out and treated with antibodies against Gr-1, CD41, CD31 and Podoplanin to stain neutrophils, platelets, endothelial and epithelial cells, respectively. Membranes were visualised via confocal microscopy (**B**). Neutrophil and platelet counts in the upper (**C**,**D**) and lower (**I**,**J**) Transwell^TM^ compartment were determined by a Sysmex haemocytometer. Neutrophils and platelets attached to the endothelium (**E**,**F**) and epithelium (**G**,**H**) were counted in microscopic images. Data are mean ± SD, n = 6, * *p* < 0.05, two-way ANOVA.

In order to investigate the transmigration defects more closely, we employed a modified transmigration assay, using an endothelial–epithelial bilayer of primary murine pulmonary cells to resemble in vivo conditions. Applying both neutrophils and platelets together, we examined the transmigration of neutrophils and their interplay with platelets during that process (Figure 2A). Neutrophils derived from S100A9^−/−^ mice migrated less efficiently than WT controls (Figure 2I). Most remained stuck in the upper compartment or adhered to the endothelial layer (Figure 2B,C,E). Interestingly, the same was observed for platelets, regardless of genotype. Close to all platelets remained in the upper compartment or on the endothelial side (Figure 2B,D,F). Barely any cells adhered to the epithelial layer (Figure 2G,H). Separate activation of platelets using thrombin did not have any effect on transmigration. These data mirrored our findings from the pneumonia model. However, they could not elucidate the role of platelets in the transmigration process, particularly since platelet activation had no consequence on the transmigration capacity.

### 3.4. Platelet–Neutrophil Complexes Are Bigger and More Abundant in S100A9^−/−^ Mice

**Figure 3:**
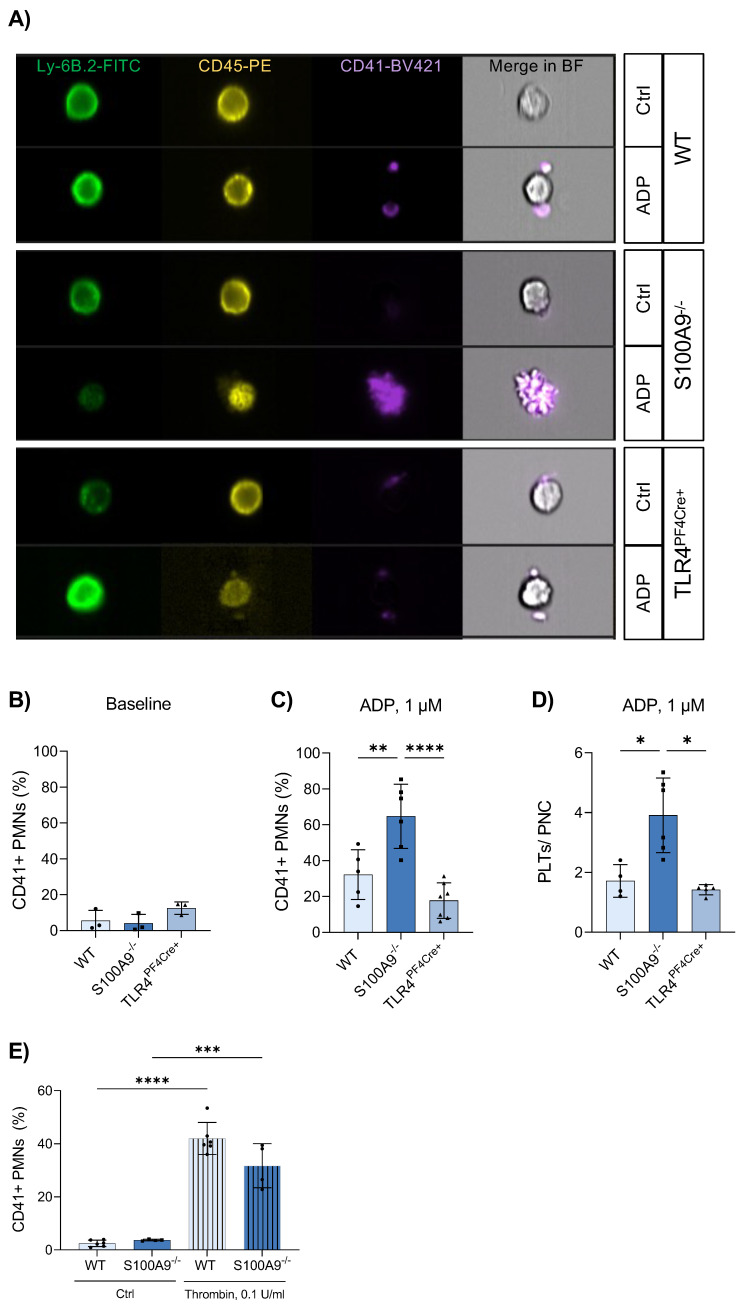
S100A9 deficiency results in an increased number of platelet–neutrophil complexes. Whole-blood samples from healthy WT, S100A9^−/−^ and TLR4^PF4Cre+^ mice were stimulated with 1 µM ADP or left untreated. Neutrophil–platelet complexes were analysed via imaging flow cytometry and identified as Ly-6B^+^CD45^+^CD41^+^ aggregates (**A**). The percentage of neutrophil–platelet complexes (CD41 + PMNs) was assessed under baseline conditions (**B**) and after ADP stimulation (**C**). The number of platelets per complex was determined manually (**D**). The formation of platelet–neutrophil complexes was simulated in vitro by coculturing platelets and bone marrow-derived neutrophils in media supplemented with 0.1 U/mL thrombin. CD41+ neutrophils (PMNs) were analysed via flow cytometry (**E**). Data are mean ± SD, n = 4–6, * *p* < 0.05, ** *p* < 0.005, *** *p* < 0.001, **** *p* < 0.0001, ordinary one-way ANOVA.

To gain a clearer picture of the interplay of platelets and neutrophils in the absence of S100A8/A9 and platelet TLR4, we employed imaging flow cytometry (Figure 3A). Imaging fresh whole blood minimised possible in vitro effects that may prevent PNC formation. Under unstimulated baseline conditions, there was no difference between the investigated phenotypes (Figure 3B). Interestingly, stimulation of the blood samples with low-dose ADP revealed significant differences in the formation of PNCs. In S100A9^−/−^ mice, there was a significant increase of both the number of PNCs (Figure 3C) and the number of platelets attached to each neutrophil (Figure 3D) compared to WT and TLR4^PF4Cre+^ mice. Extensive handling of samples disguised these effects. Furthermore, induction of PNC formation in vitro with neutrophils and platelets separately isolated from murine bone marrow and whole blood, respectively, did not reflect the in vivo findings indicating a strong in vitro effect (Figure 3E). 

**Figure 4:**
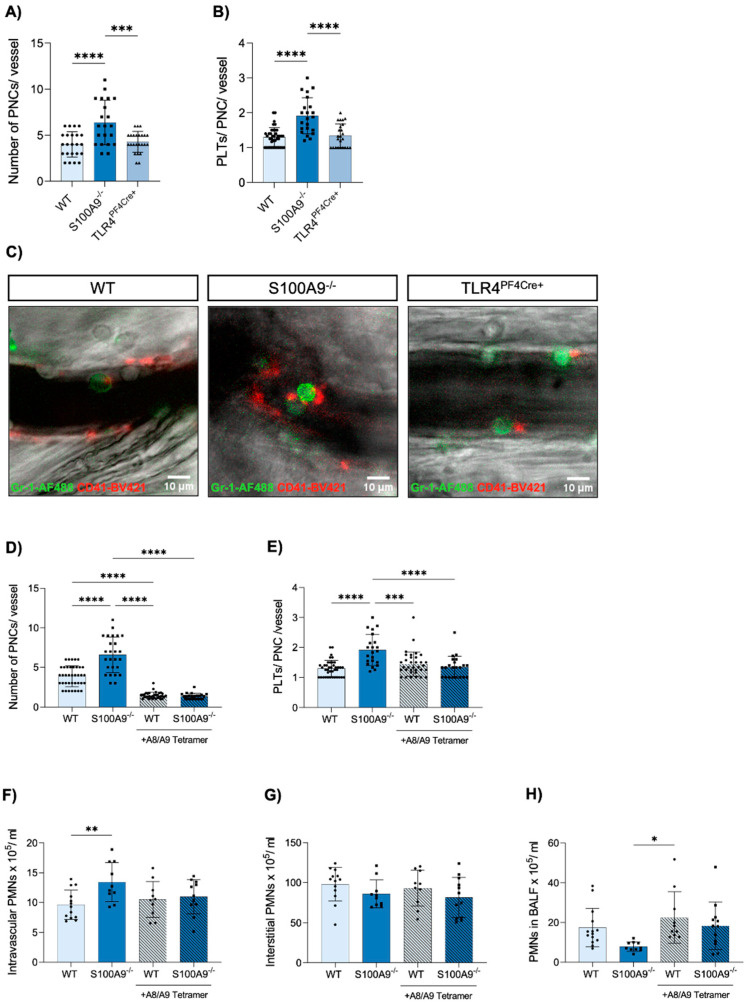
S100A9 but not platelet TLR4 deficiency increases platelet–neutrophil complexes in the cremaster muscle upon acute inflammation, an observation that is reversed by supplementation of the S100A8/A9 tetramer. (**A**) The number of platelet–neutrophil complexes in postcapillary venules in the murine cremaster muscle was analysed in WT, S100A9^−/−^ and TLR4^PF4Cre+^ mice via intravital microscopy 2 h after intrascrotal injection of TNFα, and the number of platelets per complex (**B**) were determined per vessel (n = 21–23). (**C**) Platelet–neutrophil complexes were assessed manually by the help of Gr-1-AF488 and CD41-BV421 antibody staining (representative images: green = neutrophils, red = platelets; scale bar: 10 µm). Effects of the S100A8/A9 tetramer during acute inflammation were analysed via intravital microscopy of the TNFα-inflamed cremaster and upon *K. pneumoniae* infection in WT and S100A9^−/−^ mice. S100A8/A9 tetramer (100 µg) was injected in addition to TNFα and platelet–neutrophil complexes (**D**,**E**) were investigated after 2 h. *K. pneumoniae*-challenged WT and S100A9^−/−^ mice were injected intraperitoneally with 150 µg S100A8/A9 tetramer immediately after infection, and again after 11 h. Mice were sacrificed after 22 h and neutrophil (PMN) counts in the intravascular (**F**), interstitial (**G**) and the alveolar space (**H**) were determined via flow cytometry (n = 10–12). Data are mean ± SD, * *p* < 0.05, ** *p* < 0.005, *** *p* < 0.001, **** *p* < 0.0001, ordinary one-way ANOVA or two-way ANOVA.

Our observations were not based on differences in the total number of platelets or neutrophils in murine whole blood (Supplementary Figure S3) that might cause the disparity in observed PNC formation. Examination of the expression of relevant surface markers on both platelets and neutrophils failed to deliver a satisfactory reason as to the increased rate of PNC formation in S100A9^−/−^ mice. Platelet surface receptors remained equal in expression throughout WT, S100A9^−/−^ and TLR4^PF4Cre+^, both under unstimulated and stimulated conditions (Supplementary Figure S4B,D). Equally, there were no differences in the surface marker expression between neutrophils of WT, S100A9^−/−^ and TLR4^PF4Cre^ mice that could explain an increased binding of platelets (Supplementary Figure S4A,C). In contradiction, S100A9-deficient neutrophils demonstrated reduced surface expression of CD162 under both unstimulated and stimulated conditions, which would point towards an opposite phenotype (Supplementary Figure S4A,C). Levels of fibrinogen, an important crosslinking molecule for platelets, was also not significantly changed in any of WT, S100A9^−/−^ or TLR4^PF4Cre+^ plasma samples (Figure 4E). 

### 3.5. Administration of Exogenous S100A8/A9 Tetramer Corrects Dysregulated PNC Formation

In order to confirm our findings in vivo, we employed intravital microscopy of the murine cremaster muscle and analysed PNC formation during acute inflammation. Using fluorescently labelled antibodies, we determined colocalisation of platelets and neutrophils (Figure 4C), which was defined as an interaction lasting longer than 2 s. This threshold was introduced to exclude any coincidental colocalisation during normal vessel flow. We found that in agreement with our data from imaging flow cytometry, S100A9^−/−^ mice present a significantly increased number of PNCs in their vessels as well as an increased number of platelets adherent to each neutrophil (Figure 4A,B). With this model, we demonstrate a lowered rate of transmigration concurring with increased PNC formation. 

Having shown these effects of S100A8/A9 absence on both classical neutrophil traits and platelet–neutrophil interactions, we investigated if these were due to intracellular disruptions in neutrophils alone or caused by the lack of extracellular S100A8/A9, which affects neutrophils, platelets and the endothelium. In order to differentiate between these possibilities, we administered exogenous S100A8/A9 tetramer as an additive to the TNFα application in the inflammatory murine cremaster model. We determined that the application of S100A8/A9 tetramers was enough to completely reverse the increased formation of PNCs in S100A9^−/−^ mice, where both the number of PNCs and the number of platelets per PNC were equal to WT controls after S100A8/A9 supplementation (Figure 4D,E). We were equally able to show the same effects in our acute pneumonia mouse model. Application of S100A8/A9 immediately after infection and after 11 h led to a complete loss of the differences between S100A9^−/−^ and WT mice in neutrophil recruitment to the intravascular, interstitial and alveolar compartments of the lung (Figure 4F–H). The clear effect of exogenous S100A8/A9 on neutrophil phenotypes of S100A9^−/−^ mice indicates an important role for S100A8/A9 as a mediator between the different cell types involved in the transmigration of neutrophils during inflammation.

## 4. Discussion

In this study, we demonstrate the significant role of S100A8/A9 in the transmigration of neutrophils and their interaction with platelets during inflammatory conditions. We could show that S100A8/A9-deficient mice and TLR4^PF4Cre+^ mice suffer from decreased neutrophil recruitment and bacterial killing at the site of infection in an acute *K. pneumoniae*-induced pneumonia model. The decreased transmigration rate of S100A9^−/−^ neutrophils could be replicated in an in vitro model mimicking the endothelial–epithelial bilayer of the alveolus. We could further show that both the number of PNCs as well as the number of platelets each neutrophil carried were increased in S100A9^−/−^ mice, but not TLR4^PF4Cre+^ animals, after ADP stimulation. An inflammatory model imaging the mouse cremaster muscle could confirm an increased number of PNCs, with PNCs being more abundant and larger in S100A9^−/−^ than in WT controls. These effects could not be replicated with TLR4^PF4Cre+^ mice, leading to the conclusion that platelet TLR4 is not the relevant receptor in the regulation of PNC formation. S100A8/A9 is one of the most abundant proteins in the cytosol of neutrophils [34]. In order to differentiate between intracellular and extracellular defects in the absence of S100A8/A9, we applied exogenous S100A8/A9 to our inflammatory mouse models. The abrogation of the phenotype in both lung infection and the mouse cremaster model upon application of S100A8/A9 suggests primarily an extracellular effect, and not an intracellular defect in neutrophils. 

Here, we report a strong link between S100A8/A9 deficiency and the dysregulation of PNC formation. The current paradigm dictates that platelets can bind neutrophils and activate them via direct cell–cell contacts or soluble mediators potentiating leukocyte effector functions [8]. Platelets have been reported to assist neutrophil extravasation in the past, e.g., via CD62P/PSGL-1 signalling [35,36]. In contrast, our data demonstrate a significant increase in the abundance of PNCs in S100A9^−/−^ mice with a concurrent deterioration of transmigration. To our knowledge, this is the first time that this turning point for the utility of additional platelets bound to a neutrophil is reported. In our transmigration assay, we could visualise that platelets rarely accompany the neutrophils through the endothelium, but rather detach themselves and stay either in suspension or adhere to the endothelium. This detachment of platelets from the neutrophil is dependent on the dissolution of both CD62P- and integrin-mediated interactions [37,38,39]. As we found no differences in surface marker expression on neutrophils nor platelets that could explain our findings, it is feasible that the dynamic process of integrin activation and deactivation could instead be dysregulated, resulting in larger and more abundant PNCs. Supplementation with S100A8/A9 could restore the phenotype of wild-type cells in the S100A9 knockout cells. Released S100A8/A9 heterodimers rapidly form S100A8/A9 heterotetramers upon encountering Ca^2+^, as would be the case when applied in vivo, and lose the ability to bind TLR4 [21]. After eliminating platelet TLR4 as a possible regulator of PNC formation, it is of immediate interest to determine the receptor that mediates the phenotype rescue via tetrameric S100A8/A9 in the inflammation models. It has recently been demonstrated that the tetrameric S100A8/A9 is not only the inactive form of the inflammatory heterodimer, but has the ability to exert its own functions. The tetramer is able to bind CD69 on monocytes and act in an immunoregulatory manner. Signalling via CD69 has a calming effect on monocytes and dampens inflammatory responses in disease models [22]. CD69 expression is low on neutrophils [40] but constitutively expressed on platelets [41], and appears as a likely candidate to transduce a calming effect of tetrameric S100A8/A9 to reduce PNC formation. In conclusion, we report here that S100A8/A9 deficiency leads to an irregular increase in PNCs in the blood during inflammation, correlating to impaired transmigration of neutrophils. These findings suggest a break from the paradigm that more platelets will also facilitate better extravasation. Future experiments will investigate a mechanistic link between the dysregulated PNC formation and impaired transmigration. Therefore, we will examine the role of integrin deactivation on platelets more closely, employing both functional assays and imaging techniques. It will be of special interest to determine the impact of CD69 signalling on both platelets and neutrophils during PNC formation. 

## Data Availability

All data associated with this study are available in the main text or the Supplementary Materials or are available through the corresponding author upon request.

## Supplementary Materials

The following supporting information can be downloaded at: https://www.mdpi.com/article/10.3390/cells11233944/s1.
