# Supplementary material for: Role of S100A8/A9 in Platelet–Neutrophil Complex Formation during Acute Inflammation
Source: Cells. 2022 Dec 6;11(23):3944. doi: 10.3390/cells11233944 (PMC9738100; doi:10.3390/cells11233944)

Supplemental Materials

Supplemental Figure S1: **Infection of S100A9 and platelet TLR4 deficient mice with *K. pneumoniae* results in elevated plasma cytokine levels.** S100A9$^{-/-}$ and TLR4$^{PF4Cre+}$ mice were challenged with 40 x 10^6 *K. pneumoniae* via intratracheal injection to induce acute pneumonia. Mice were sacrificed after 22 h and blood was drawn via cardiac puncture into heparinised syringes. Blood was centrifuged and plasma was collected. ProcartaPlex Panel analysis of 36 cytokines was performed with plasma samples. Significant (dark blue) and nearly significant (light blue) differences are highlighted. n=4, ordinary one-way ANOVA.

Supplemental Figure S2: **Platelets do not express endogenous S100A8 and S100A9 but slightly respond to S100A8/A9 stimulation.** Platelets and bone marrow-derived neutrophils from WT, S100A9$^{-/-}$ and TLR4$^{PF4Cre+}$ were lysed and western blotting was performed to analyse endogenous S100A8 and S100A9 protein levels **(A)**. Platelets were isolated from TLR4$^{PF4Cre-}$ and TLR4$^{PF4Cre+}$ mice via repetitive slow-speed centrifugation. TLR4/MD2 expression was measured via flow cytometry (grey = isotype control, blue = TLR4$^{PF4Cre-}$, orange = TLR4$^{PF4Cre+}$) **(B)**. Platelet activation upon S100A8/A9N70AE79A (40 µg/ml) stimulation was determined by flow cytometry by analysing CD62P mobilisation and GPIIbIIIa activation. **(C)** Platelets were identified as CD41^{+} events (orange = unstained control, pink = CD41^{+} population, light red = unstimulated control, dark red = S100A8/A9 stimulation).

Supplemental Figure S3: **White blood cell and platelets counts do not differ between WT, S100A9$^{-/-}$ and TLR4$^{PF4Cre+}$ mice.** Blood was drawn from WT, S100A9$^{-/-}$ and TLR4$^{PF4Cre+}$ mice by cardiac puncture into a heparinised syringe. PLT **(A)**, neutrophil **(B)**,

total white blood cell **(C)**, lymphocytes **(D)**, monocytes **(E)** and eosinophil **(F)** counts were determined in a Sysmex haemocytometer.

Supplemental Figure S4: **Surface expression analyses of proteins mediating physical platelet-neutrophil contacts.** Blood was drawn from WT, S100A9$^{-/-}$ and TLR4$^{PF4Cre+}$ mice by cardiac puncture into an ACD-coated syringe. Surface marker expression of CD11a, CD11b, CD40, CD162 and TREM-1 was identified on whole blood neutrophils with and without ADP (1 µM) stimulation **(A, C)**, whereas expression of GP1bα, activated GPIIbIIIa, CD40L, CD62P and CD102 was identified on platelets in the same samples **(B, D)**. Plasma samples from WT, S100A9$^{-/-}$ and TLR4$^{PF4Cre+}$ mice were analysed by ELISA for differences in fibrinogen levels **(E)**.

Suppl. Figure S1

Analyte	S100A9$^{-/-}$ - WT	TLR4$^{PF4Cre+}$ - WT
GM/CSF	0.0104	0.1373
IFNγ	0.0315	0.5471
IL-1b	0.0082	0.5500
IL-12p70	0.0179	0.3863
IL-13	0.0393	0.6821
IL-18	0.0170	0.3241
IL-2	0.0708	0.8120
IL-4	0.0249	>0.9999
IL-5	0.0164	0.7828
IL-6	0.0846	0.8392
TNFα	0.0023	0.0691
CXCL5	0.9635	0.8080
CSF3	0.1339	0.0233
IFNα	>0.9999	>0.9999
IL-1a	0.8929	0.9695
IL-15	>0.9999	>0.9999
IL-28	>0.9999	>0.9999
IL-3	>0.9999	>0.9999
IL-31	>0.9999	>0.9999
LIF	0.0957	0.4007
M-CSF	>0.9999	>0.9999
IL-10	0.0037	0.3856
IL-17a	0.0454	>0.9999
IL-22	0.0128	0.9428
IL-23	0.5256	0.1704
IL-27	0.0855	0.9307
IL-9	0.3264	0.3264
CCL11	0.1253	0.7839
CXCL1	0.0607	0.5397
CXCL10	0.0168	0.6816
CCL2	0.0082	0.1222
CCL7	0.0006	0.2460
CCL3	0.0006	0.2460
CCL4	0.0861	0.9339
CXCL2	0.0308	0.5701
CCL5	0.0014	0.4849

Significantly upregulated

Nearly significant

Suppl. Figure S2

A)

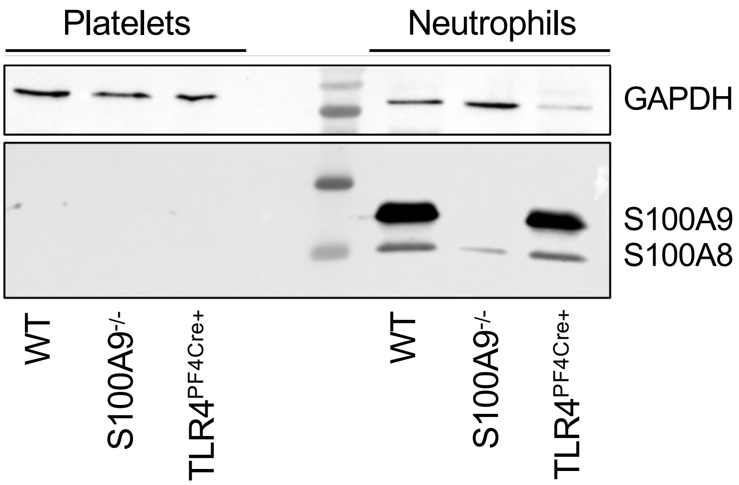

B)

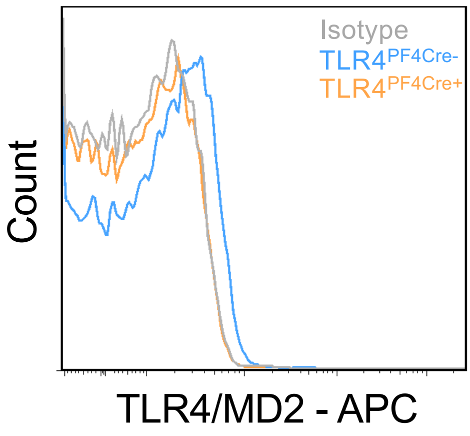

C)

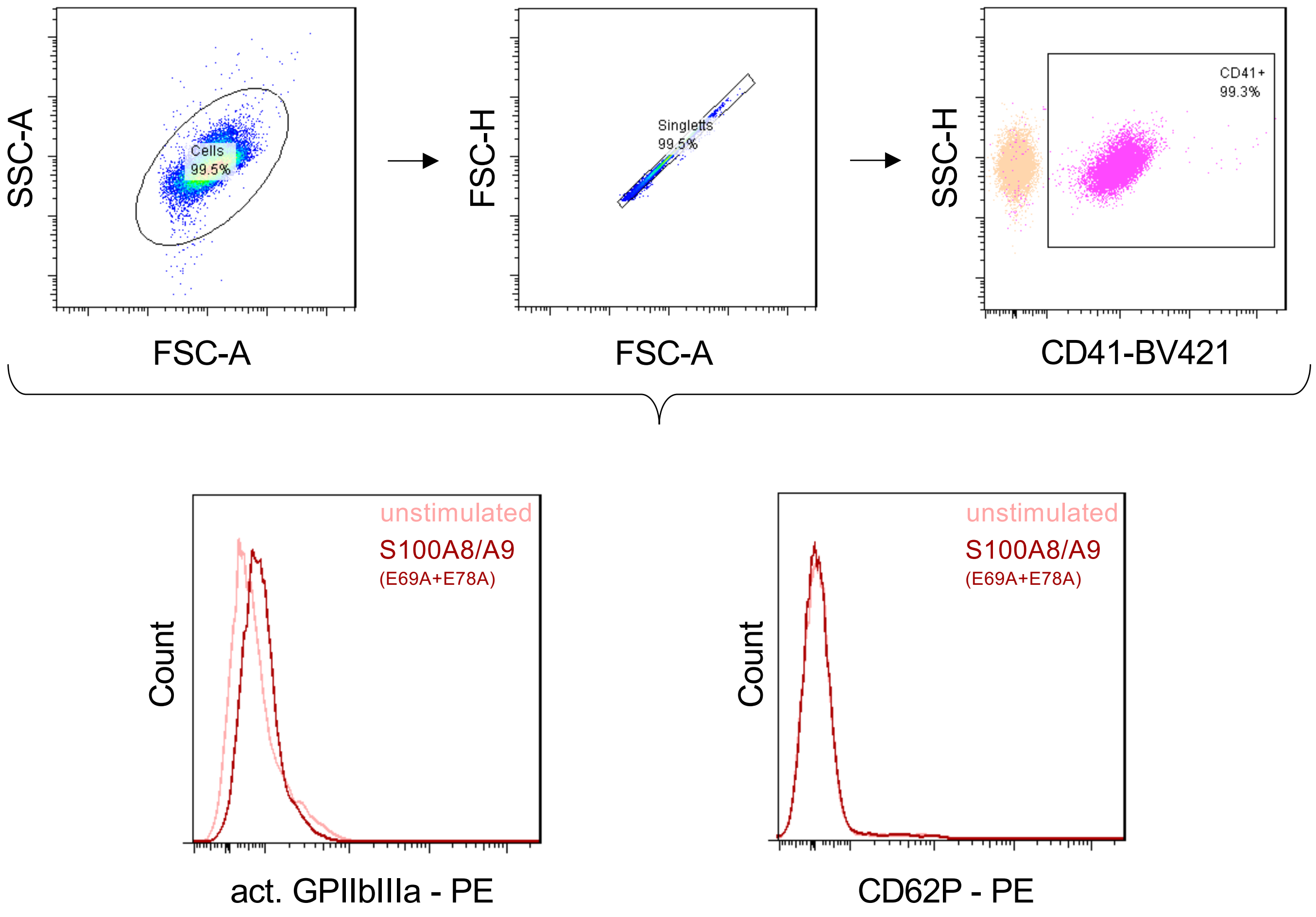

Suppl. Figure S3

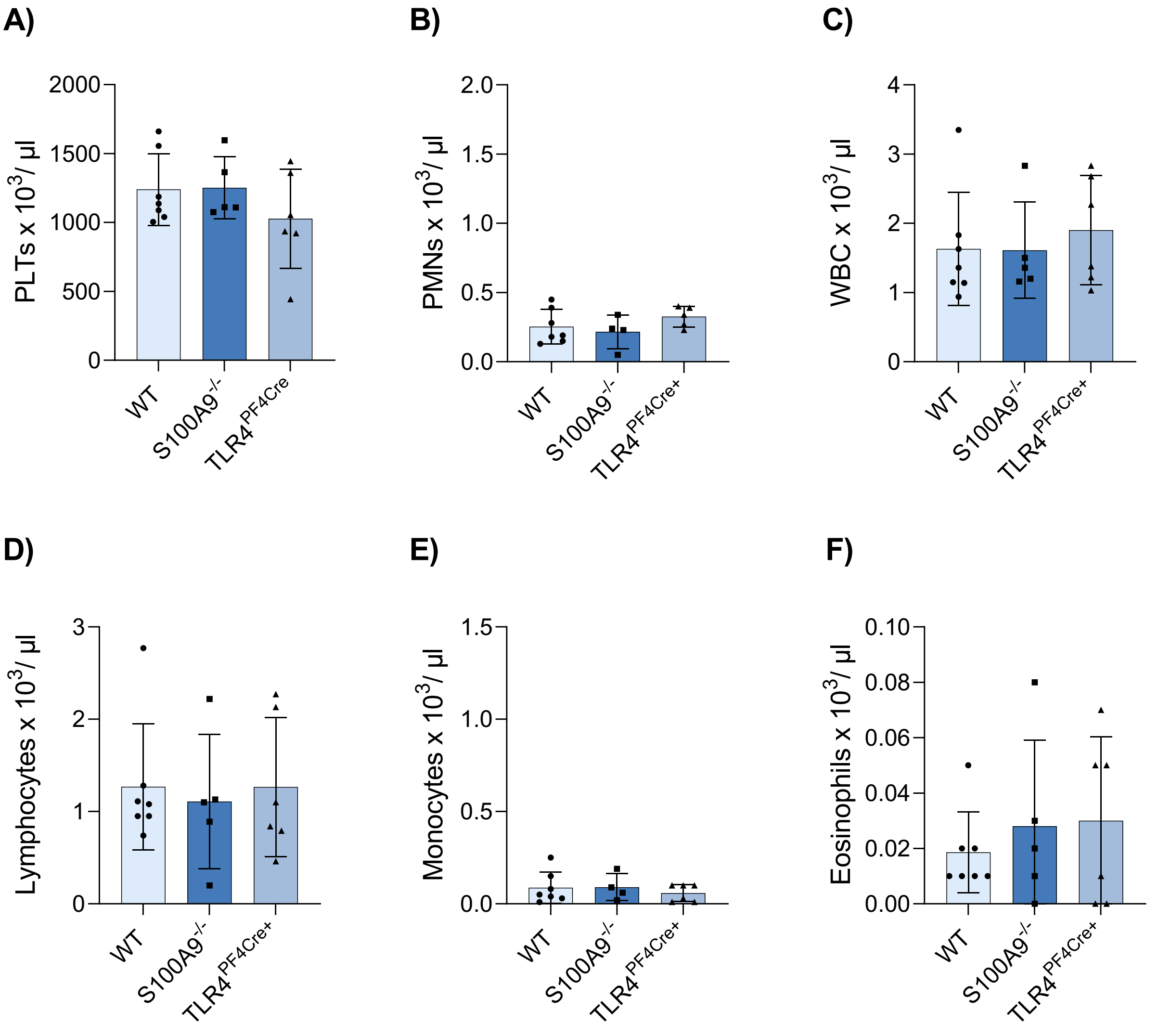

Suppl. Figure S4

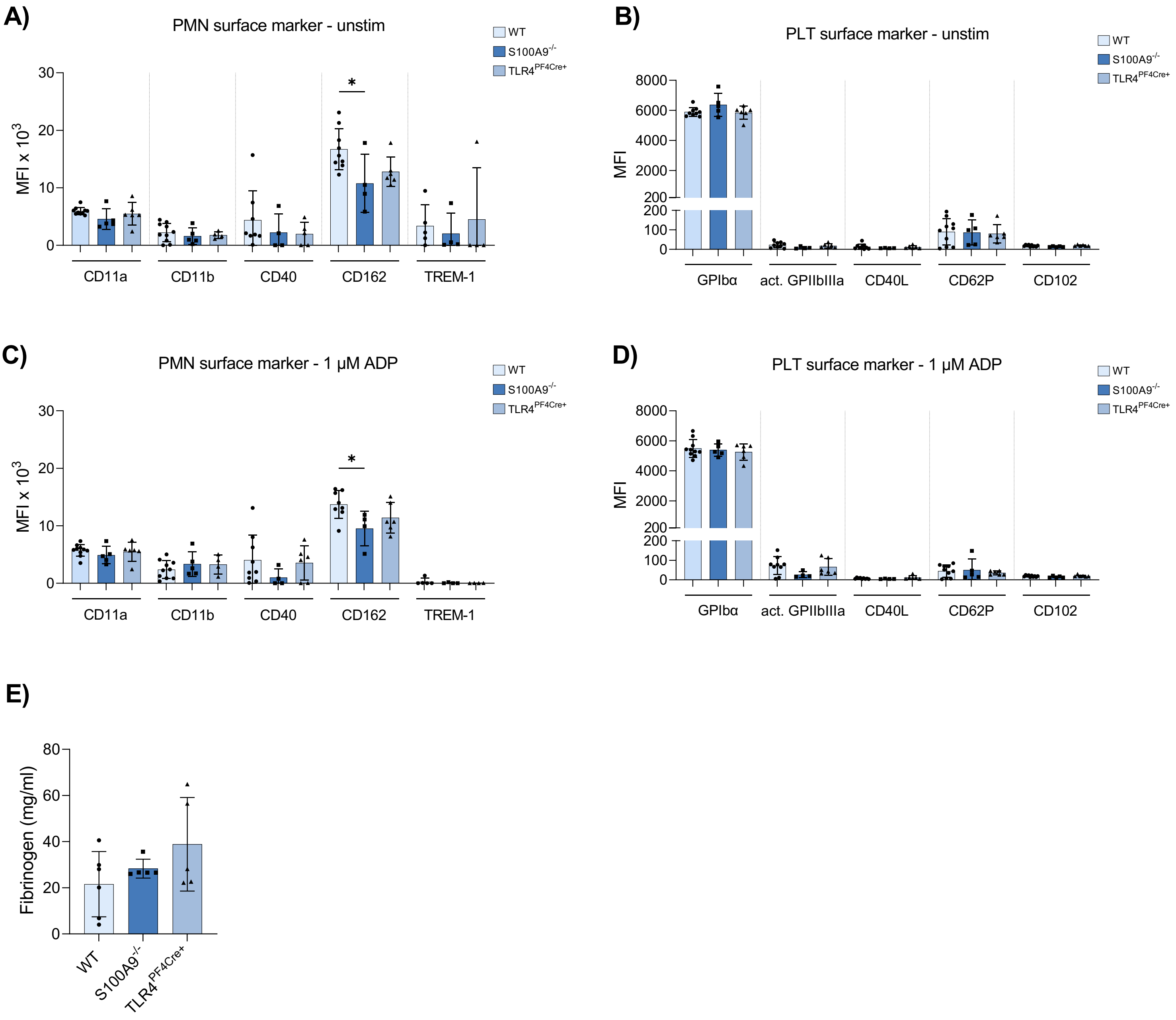

Supplement: Supplementary file 1 [file cells-11-03944-s001.zip › cells-2045963-supplementary.pdf]
